# Single-nucleus chromatin accessibility reveals intratumoral epigenetic heterogeneity in IDH1 mutant gliomas

**DOI:** 10.1186/s40478-019-0851-y

**Published:** 2019-12-05

**Authors:** Ruslan Al-Ali, Katharina Bauer, Jong-Whi Park, Ruba Al Abdulla, Valentina Fermi, Andreas von Deimling, Christel Herold-Mende, Jan-Philipp Mallm, Carl Herrmann, Wolfgang Wick, Şevin Turcan

**Affiliations:** 1 Neurology Clinic and National Center for Tumor Diseases, University Hospital Heidelberg, INF 460, 69120 Heidelberg, Germany; 2 Institute Cochin, INSERM U1016, UMR 8104 CNRS, Faculté René Descartes, 24 rue du Faubourg St Jacques, 75014 Paris, France; 3 Single-cell Open Lab, German Cancer Research Center (DKFZ), Heidelberg, Germany; 4 Institute of Research, Development and Innovation in Healthcare Biotechnology in Elche- IDiBE, Elche, Spain; 5 Division of Experimental Neurosurgery, Department of Neurosurgery, University of Heidelberg, INF 400, D-69120 Heidelberg, Germany; 6 Department of Neuropathology, Institute of Pathology, University of Heidelberg, INF 224, D-69120 Heidelberg, Germany; 7 Clinical Cooperation Unit Neuropathology, German Consortium for Translational Cancer Research (DKTK), German Cancer Research Center (DKFZ), Heidelberg, Germany; 8 Division of Chromatin Networks, German Cancer Research Center (DKFZ) and BioQuant, Heidelberg, Germany; 9 Health Data Science Unit, Medical Faculty University Heidelberg and BioQuant, 69120 Heidelberg, Germany; 10 Clinical Cooperation Unit Neurooncology, German Cancer Consortium (DKTK), German Cancer Research Center (DKFZ), Heidelberg, Germany

## Abstract

The presence of genome-wide DNA hypermethylation is a hallmark of lower grade gliomas (LGG) with isocitrate dehydrogenase (IDH) mutations. Further molecular classification of IDH mutant gliomas is defined by the presence (IDHmut-codel) or absence (IDHmut-noncodel) of hemizygous codeletion of chromosome arms 1p and 19q. Despite the DNA hypermethylation seen in bulk tumors, intra-tumoral heterogeneity at the epigenetic level has not been thoroughly analyzed. To address this question, we performed the first epigenetic profiling of single cells in a cohort of 5 gliomas with IDH1 mutation using single nucleus Assay for Transposase-Accessible Chromatin with high-throughput sequencing (snATAC-seq). Using the Fluidigm HT IFC microfluidics platform, we generated chromatin accessibility maps from 336 individual nuclei, and identified variable promoter accessibility of non-coding RNAs in LGGs. Interestingly, local chromatin structures of several non-coding RNAs are significant factors that contribute to heterogeneity, and show increased promoter accessibility in IDHmut-noncodel samples. As an example for clinical significance of this result, we identify *CYTOR* as a poor prognosis factor in gliomas with IDH mutation. Open chromatin assay points to differential accessibility of non-coding RNAs as an important source of epigenetic heterogeneity within individual tumors and between molecular subgroups. Rare populations of nuclei that resemble either IDH mutant molecular group co-exist within IDHmut-noncodel and IDHmut-codel groups, and along with non-coding RNAs may be an important issue to consider for future studies, as they may help guide predict treatment response and relapse.

A web-based explorer for the data is available at shiny.turcanlab.org.

## Introduction

Isocitrate dehydrogenase 1 (IDH1), and to a lesser extent, its mitochondrial homolog, IDH2, are mutated in a majority of adult lower grade gliomas (LGGs) [46]. IDH proteins normally serve as the core metabolic enzymes in the citric acid cycle and convert isocitrate to α-ketoglutarate (αKG). The most common IDH1 mutation in gliomas (IDH1 R132) occurs in the catalytic domain of IDH1 and confers the ability to produce 2-hydroxyglutarate (2-HG) [7]. 2-HG competitively inhibits enzymes that use αKG as a cofactor, such as TET family of enzymes and Jmj-C domain containing histone demethylases, leading to DNA hypermethylation, and aberrant methylation of a number of histone marks along with impaired differentiation leading to an expansion of stem/progenitor cells [10, 27, 40]. Gliomas with IDH mutation exhibit global DNA hypermethylation and are subdivided into two distinct molecular subgroups: IDHmut-codel (hemizygous co-deletion of chromosome arms 1p/19q) and IDHmut-noncodel (without co-deletion of 1p/19q) gliomas [4]. While transcriptional heterogeneity at the single cell level and longitudinal alterations in the bulk epigenomes of IDH mutant gliomas have been investigated, little is known about intratumoral epigenetic heterogeneity at the single cell level [16, 39, 43]. To address this question, we interrogated the accessible chromatin at the individual cell level in gliomas with IDH mutation using single nucleus Assay for Transposase-Accessible Chromatin with high-throughput sequencing (snATAC-seq) on a subset of 5 patient samples.

Using the Fluidigm microfluidics platform, we established a biologically-relevant analysis to overcome the technical limitation and high background noise associated with snATAC-seq. We identified heterogeneity in promoter accessibility within and between IDHmut-codel and IDHmut-noncodel samples. Interestingly, our results indicate differential accessibility of non-coding RNAs such as the CYTOR locus that exhibits a profound increase in promoter accessibility within IDHmut-noncodel tumors. Furthermore, we identify *CYTOR* as a poor prognosis factor in gliomas with IDH mutation. Overall, our results point to differential accessibility of non-coding RNAs as an important source of epigenetic heterogeneity within individual tumors and between molecular subgroups. The molecules identified are promising targets for future molecular research.

## Results

Our cohort was primarily composed of WHO grade II gliomas, with the exception of one WHO grade III IDHmut-noncodel glioma, and all tumors harbored an IDH1 R132 mutation (Table 1). We used the Fluidigm HT IFC microfluids platform to perform snATAC-seq. We started with 7 tumors samples from glioma patients, but only 5 samples passed the quality control. We used stringent cut-offs and excluded unreliable cells, obtaining DNA accessibility maps for a total of 336 cells (Additional file 1: Table S1). Our analysis indicated a previously undescribed presence of identical reads leaking across specific rows and columns of the microfluidic chamber (Additional file 2: Figure S1a, b). We reasoned that these cross-contaminating reads were unlikely to be biologically relevant and may indicate an inherent technical issue with the microfluidics platform. To overcome this technical noise, we removed the leaky reads from all samples (Additional file 2: Figure S1c-f). Overall, we obtained high-quality DNA accessibility maps from 145 cells from 3 IDHmut-noncodel gliomas (Astro1, Astro2, Astro3), and 191 cells from 2 IDHmut-codel gliomas (Oligo1, Oligo2), with a total of 336 cells (Additional file 1: Table S1).

**Table 1:** Patient samples used for snATAC-seq

Pseudonym	IDH mutation	Histology	Grade	Sample ID
NCH5526	IDH1 R132S	Astrocytoma	Grade II	Astro1
NCH6015	IDH1 R132H	Astrocytoma	Grade II	Astro2
NCH5559	IDH1 R132H	Astrocytoma	Grade III	Astro3
NCH5540	IDH1 R132H	Oligodendroglioma	Grade II	Oligo1
NCH5699	IDH1 R132H	Oligodendroglioma	Grade II	Oligo2

**Fig. 1:**
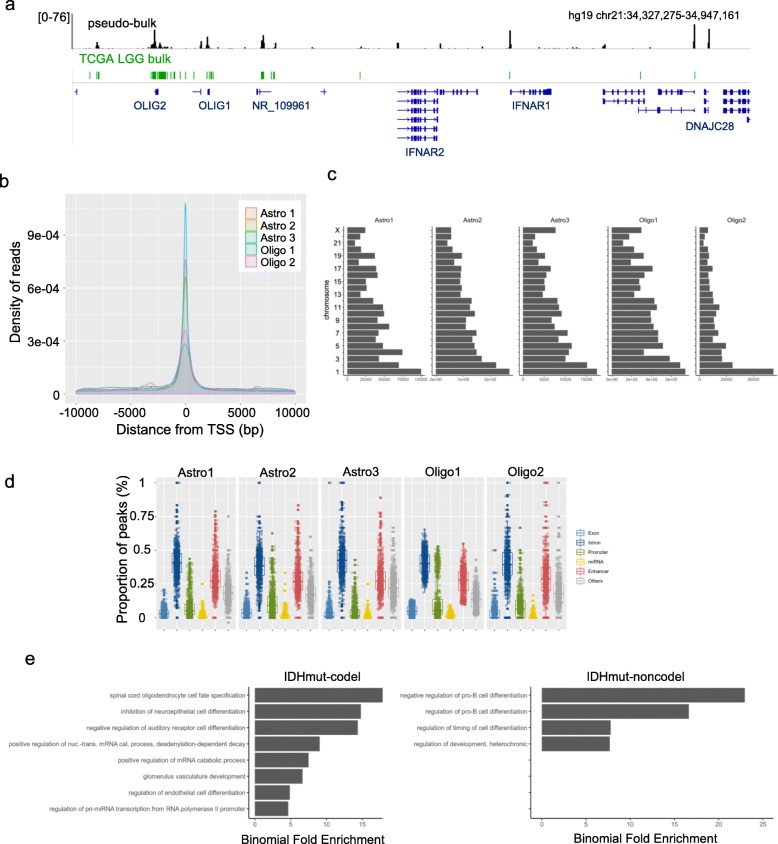
IDH1 mutant LGGs exhibit differential chromatin accessibility. **a** Comparison of peaks called from pseudo-bulk snATAC-seq data (black) and bulk ATAC-seq on TCGA LGG data (green), **b** Density of read distribution for all samples around transcription start sites (TSS), **c** Distribution of the number of reads on all chromosomes for each sample, **d** Proportion of peaks that fall within each annotation genome class for each sample, **e** Gene set enrichment analysis for the enhancer regions of IDHmut-codel (left) and IDHmut-noncodel (right) samples

We applied a pipeline (HOMER), commonly used for peak calling from ChIP-seq data, to call peaks in our snATAC-seq datasets on both pseudo-bulk and single nuclei level. Peaks called from pseudo-bulk profiles of the snATAC-seq data closely resembled bulk ATAC-seq data obtained from IDH mutant TCGA LGG samples (Fig. 1a) [6]. For all samples, majority of the reads centered around the transcription start sites (TSS) (Fig. 1b). Reads were distributed evenly and proportionally across chromosomes (Fig. 1c). The majority of peaks mapped to intronic and enhancer regions (Fig. 1d). We used the GREAT toolbox to assess whether the enhancer regions were enriched for any particular gene sets. Our analysis indicated that enhancers in IDHmut-noncodel samples were enriched in gene sets associated with suppression of pro-B cell differentiation and development. IDHmut-codel enhancer regions were enriched for gene sets associated with mRNA regulation, spinal cord oligodendrocyte cell fate and inhibition of neuroepithelial differentiation (Fig. 1e).

**Fig. 2:**
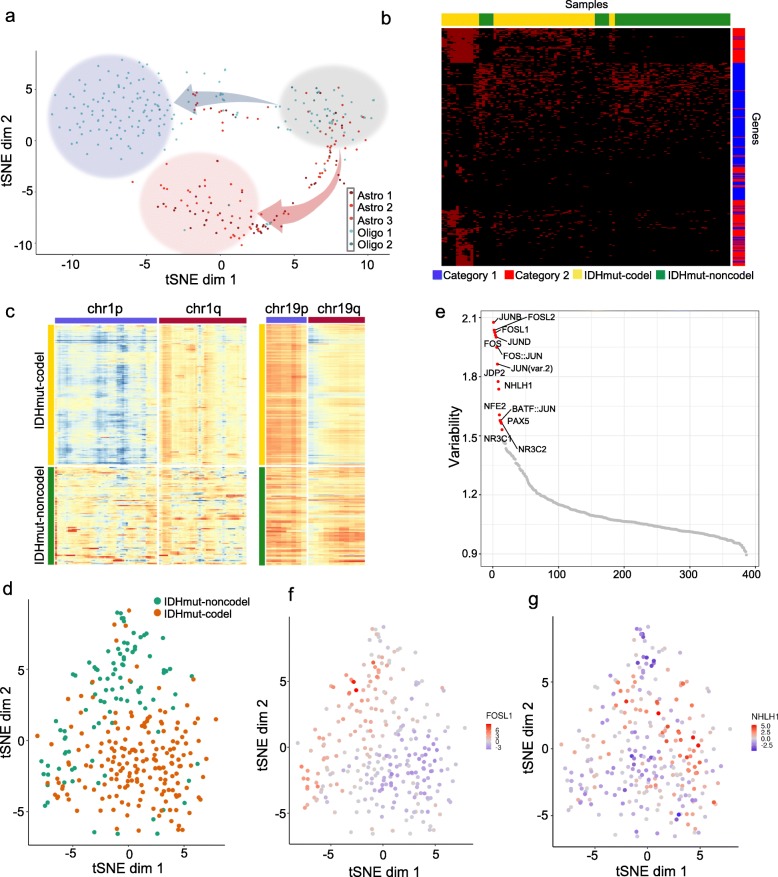
Heterogeneity in accessibility of transcription factors within gliomas with IDH mutation. **a** t-SNE plot of snATAC-seq data, **b** Accessibility heatmap of the markers that separate the two blue and red states, as shown in (**a**), category 1 indicates the blue cluster, and category 2 indicates the red cluster, **c** Normalized ATAC-seq coverage for the single nuclei from IDHmut-codel and IDHmut-noncodel samples for chromosome 1 (left) and chromosome 19 (right). The 1p/19q co-deletion in a subset of cells is seen when comparing the IDHmut-codel to IDHmut-noncodel nuclei, **d** t-SNE plot of the transcription factor variability scores obtained from chromVAR, **e** Ranked plot of variability scores for 386 TF motifs (chromVAR), **f** t-SNE plot of variability scores for FOSL1 (chromVAR), **g** t-SNE plot of variability scores for NHLH1 (chromVAR)

Next, to reduce noise, we decided to limit our analysis to accessible peaks identified from bulk LGGs. To achieve this, we overlapped the peaks from our snATAC-seq data with the peaks called from bulk ATAC-seq of TCGA LGG samples [6] (Additional file 2: Figures S2 and S3). We used these overlapping peaks for the remainder of our study. Subsequently, we applied t-distributed stochastic neighbor embedding (t-SNE) to reduce the dimensionality of the snATAC-seq data. The t-SNE mapping showed three clusters, branched along two trajectories (upper and lower), coalescing on two clusters as indicated with red and blue circles (Fig. 2a). Approximately 20% of the cells could not be classified and remained in a gray zone due to lack of sufficiently specific peaks, while the upper and lower clusters were distinct, and almost entirely consisted of cells from a particular molecular subtype (Fig. 2a). Heatmap of open promoters unique to each trajectory indicates variable accessibility within or between IDHmut-codel or IDHmut-noncodel samples (Fig. 2b).

To determine whether 1p/19q codeletion can be detected from the snATAC-seq data, we inferred large-scale copy number alterations in these chromosomes by averaging ATAC-seq coverage for each nuclei. This analysis indicated decreased coverage for both 1p and 19q arms relative to 1q and 19p in IDHmut-codel tumors, revealing molecular evidence in the snATAC-seq data for the codeletion pattern in these samples (Fig. 2c).

### Differential accessibility of transcription factors

To determine whether chromatin accessibility within transcription factor (TF) binding sites differ, we applied chromVAR to identify highly variable TF motifs [33] (Additional file 3: Table S2, Additional file 2: Figure S4a). We used the TF z-scores to visualize the snATAC-seq data using t-SNE. Similar to Fig. 2a, these features also separated the data into two states, driven by differences in molecular subtype (Fig. 2d). A heatmap visualization showed heterogeneity of TF accessibility within samples belonging to a specific molecular subgroup (Additional file 2: Figure S4b), We identified 14 TFs that exhibited high variability (> 1.5) (Fig. 2e). The highest variability was observed by differences in two major families of AP-1 transcription factors: JUN and FOS that are expressed downstream of the mitogen-activated protein kinase (MAPK) signaling cascades (Fig. 2f). A subgroup of IDHmut-codel samples also displayed differential NHLH1 accessibility (Fig. 2g). NHLH1 is required for the formation of pre-cerebellar neurons in the hindbrain [34]. In addition, PAX5 showed differential accessibility in a subset of cells (Additional file 3: Table S2). PAX5 plays an essential role for normal development of the midbrain and cerebellum [41]. Moreover, several known oncogenic transcription factors, such as the ETS family, that are aberrantly activated in a majority of IDHmut-codel tumors, also exhibited significant variability within IDHmut-codel and IDHmut-noncodel tumors [19].

### Non-coding RNAs and transcription factors are differentially accessible in gliomas with IDH mutation

**Fig. 3:**
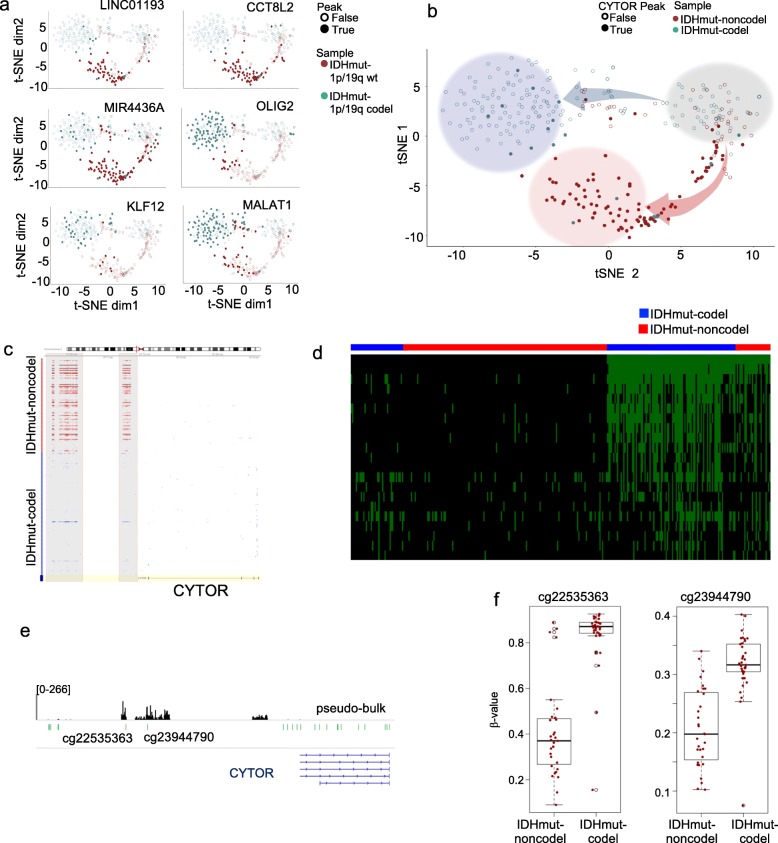
CYTOR promoter is hypermethylated and not accessible in a majority of nuclei in IDHmut-codel gliomas. **a** Promoter accessibility for several non-coding RNAs (LINC001193, CCT8L2, MIR4436A, and MALAT1), and coding RNAs (KLF12, OLIG2) are indicated on the t-SNE plot. Maroon, IDHmut-noncodel; Teal, IDHmut-codel; False, no peak detected; True peak present, **b** t-SNE plot indicating nuclei with open CYTOR promoter. Gray category includes nuclei that were not well-distinguished. In the upper trajectory, the blue arrow indicates branching of IDHmut-codel samples, and the blue category is the top of the branch. In the lower trajectory, the red arrow indicates a longer branch that largely includes IDHmut-noncodel samples, and the red circle is the top part of the branch. Maroon, IDHmut-noncodel; Teal, IDHmut-codel; False, no peak detected; True peak present, **c** Heatmap shows the reads within and upstream of the CYTOR transcription start site. While most of the IDHmut-noncodel nuclei (red) have open chromatin, only a small percentage of IDHmut-codel nuclei have the same pattern (blue), **d** Heatmap of CYTOR peaks and top correlated peaks. It shows that most of astrocytoma cells and a small population of oligodendroglioma cells Have an open chromatin peak at CYTOR promotor and other correlating peaks, **e** Overlap of CpG locations on the EPIC array (green) with the peaks from pseudo-bulk snATAC-seq data (black) within and upstream of the CYTOR locus, **f** Box-plot showing methylation b-values for the overlapping probes, cg22535363, and cg23944790 for IDHmut-noncodel and IDHmut-codel tumors

We identified IDHmut-noncodel and IDHmut-codel specific peaks near the promoters of several non-coding RNAs. These included LINC01193, CCT8L2 and MIR4436A, that exhibited increased chromatin accessibility in IDHmut-noncodel samples, and MALAT1 with increased chromatin accessibility in IDHmut-codel samples (Fig. 3a). MALAT1 and LINC01193 are known to have functions in cancer [12, 47]. We also identified accessible promoters enriched in IDHmut-codel samples, including OLIG2, and KLF12 (Fig. 3a, Additional file 4: Table S3). Of note, OLIG2, an essential transcription factor for inducing oligodendrocyte development, has an open promoter in the majority of IDHmut-codel nuclei, whereas only a few nuclei in IDHmut-noncodel samples harbor an accessible OLIG2 promoter (Fig. 3a).

### *CYTOR* as an example for differentially enriched non-coding RNA with clinical significance

Our snATAC-seq data pointed to a striking difference in accessibility around and within the CYTOR promoter. The t-SNE visualization revealed accessible CYTOR promoter along the lower branch trajectory, which is largely defined by IDHmut-noncodel samples (Fig. 3b). Specifically, 70% (101/145) of IDHmut-noncodel samples, and 10.5% (20/191) of IDHmut-codel samples had an open CYTOR promoter (Fig. 3c). We searched for co-accessible regions with the CYTOR promoter in the combined snATAC-seq dataset and identified 50 correlated regions (Pearson > 0.2) (Fig. 3d, Additional file 5: Table S4). Interestingly, one of the correlated regions was the promoter of ID2, a transcriptional regulator that supports a pro-survival role in malignant gliomas by inactivating VHL [22, 48]. CYTOR (LINC00152) is a long non-coding RNA that regulates cytoskeleton and plays an oncogenic role in several cancers, including colorectal cancer, and gastric cancer [45, 49]. CYTOR is associated with poor prognosis and is upregulated in diffuse gliomas, and higher grade IDH wild-type gliomas and glioblastomas [31, 52].

To assess the methylation state of the CYTOR locus, we utilized data from 68 gliomas with IDH mutation profiled using the Illumina Infinium MethylationEPIC BeadChip (EPIC) arrays as a part of the DNA-methylation based classification efforts of central nervous system tumor entities [5]. The methylation data included samples from 31 IDHmut-noncodel and 37 IDHmut-codel. We identified 10,634 differentially methylated sites between the two IDH molecular subgroups at a q-value < 0.001 and absolute β-value> 0.1 (Additional file 6: Table S5). To determine whether there was an inverse correlation between accessible chromatin and DNA methylation upstream of CYTOR TSS, we overlapped the location of the EPIC array probes with the snATAC-seq peaks called from pseudo-bulk regions. This analysis identified two overlapping CpG probes: cg22535363, and cg23944790 (Fig. 3e). Both of these probes were significantly hypermethylated in IDHmut-codel samples when compared to IDHmut-noncodel samples (Fig. 3f), further suggesting the limited chromatin accessibility of CYTOR in IDHmut-codel gliomas.

**Fig. 4:**
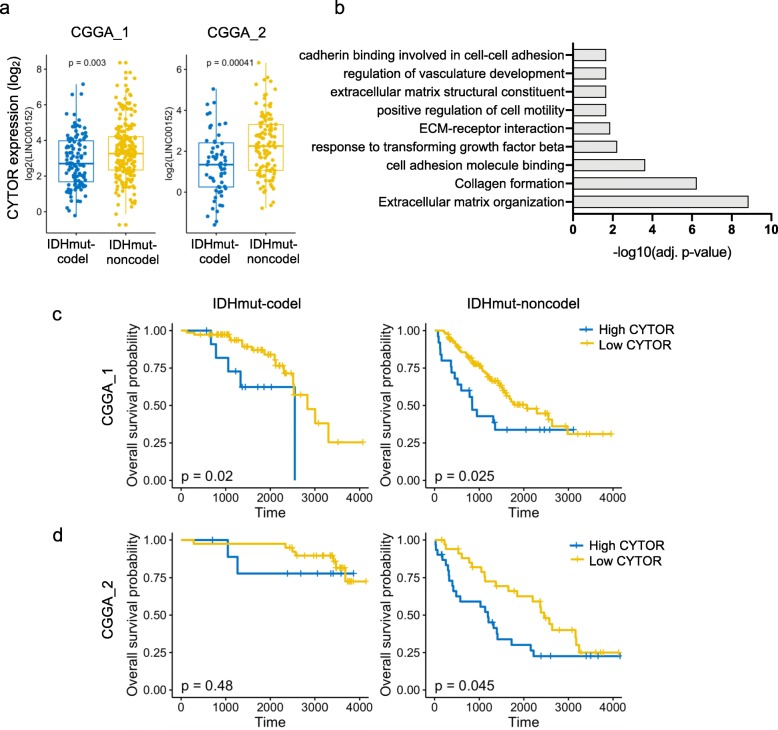
*CYTOR* is associated with poor overall survival in gliomas with IDH mutation. **a** Boxplot indicates *CYTOR* expression in IDHmut-codel (blue) and IDHmut-noncodel samples (yellow) in the Chinese Glioma Genome Atlas (CGGA_1, and CGGA_2), **b** Functional enrichment of genes correlating with *CYTOR* in both CGGA cohorts. *Adj. p-value, adjusted p-value*, **c** Kaplan-Meier plots for IDHmut-codel (left), and IDHmut-noncodel (right) gliomas indicating survival between samples with high and low *CYTOR* expression in CGGA_1 dataset, **d** Kaplan-Meier plots for IDHmut-codel (left), and IDHmut-noncodel (right) gliomas indicating survival between samples with high and low *CYTOR* expression in CGGA_2 dataset

Next, we wondered whether these differences in CYTOR at the DNA level were reflected at a transcriptional level. To answer this question, we analyzed bulk RNA-seq data from the Chinese Glioma Genome Atlas (CGGA), which includes two datasets with 693 (CGGA-1) and 325 (CGGA-2) samples, respectively. We restricted our analyses to IDH mutant gliomas (WHO Grade II-IV) within the CGGA data, which included 258 CGGA-1 samples and 152 CGGA-2 samples. *CYTOR* expression was significantly higher in IDHmut-noncodel samples compared to IDHmut-codel samples in both datasets (Fig. 4a). Overlap of genes positively correlated with CYTOR in both datasets (198 genes in CGGA-1, and 541 genes in CGGA-2) (Pearson > 0.5) revealed 101 genes with high correlation to *CYTOR* (Additional file 7: Table S6) Interestingly, these genes were significantly enriched for several pathways including regulation of migration, regulation of vasculature development, and collagen formation (Fig. 4b, Additional file 8: Table S7). Finally, we asked whether *CYTOR* expression was correlated with overall survival in gliomas with IDH mutation (WHO Grade II-III). We identified the maximal cut-off point and classified each tumor by high- or low- CYTOR expression. Comparison of IDHmut-noncodel and IDHmut-codel samples revealed that high CYTOR expression was associated with poor overall survival prognosis in both CGGA and TCGA data sets (Fig. 4c, d, Additional file 2: Figure S6a, b).

### Discussion

Gliomas with IDH mutation exhibit global DNA hypermethylation. While previous studies have investigated heterogeneity at the transcriptional level, it is unclear whether these tumors harbor epigenetic heterogeneity at the individual cell level. To our knowledge, our study is the first description of chromatin accessibility of gliomas with IDH mutation using single nucleus ATAC-seq. Using conservative cutoff values, and by relying on overlapping peaks from bulk ATAC-seq from IDH mutant glioma patients samples, we were able to reduce noise and constrain our analysis to biologically significant regions. We observed differential accessibility of several transcription factor binding sites, within and between IDHmut-codel and IDHmut-noncodel samples. Accessibility of DNA binding sites for JUN or FOS were highly variable, and interestingly were accessible to a much lower extent in IDHmut-codel tumors. Activity of AP-1, the transcriptional activator composed of members of the Jun and Fos families are regulated by MAPK signaling, and control proliferation and apoptosis [35]. Sequence binding-specific sites of AP-1 transcription factors are reported to be hypomethylated targets in glioblastoma with poor clinical outcome and low glioma CpG Island Methylator Phenotype (G-CIMP) [8]. Several transcription factors, such as NHLH1 and PAX5 with known roles in neurodevelopment also exhibit differential accessibility among IDHmut-codel and IDHmut-noncodel samples. Although this remains to be determined, it is possible that these accessible TF motifs are vestiges of cell of origin for these tumors. This is also suggested by gene set enrichment analysis which showed significant enrichment for neural lineage differentiation pathways, particularly for IDHmut-codel gliomas. In addition, we identify that ETS transcription factors display heterogeneity with increased variability in accessibility in IDHmut-noncodel tumors. The ETS/AP-1 transcription factors regulate a RAS-responsive gene expression program. PEA3 subfamily of ETS proteins (ETV1, ETV4, ETV5) amplify transcriptional signals when RAS/MAPK signaling pathway is active, and abolishing *Ets* activity leads to a block in glioma initiation [2, 14].

Our data show that majority of IDHmut-codel nuclei, and only a subset of IDHmut-noncodel nuclei harbor an open chromatin within the promoter of OLIG2. Expression of OLIG2 is restricted to the central nervous system and determines oligodendrocyte and astrocyte fate determination in the developing brain [28, 50, 51]. It is ubiquitously expressed in gliomas, and was identified as one of the core transcription factors that can reprogram differentiated GBM cells into glioma stem cells [25, 26, 37]. Our results indicate that *CYTOR* could be of importance in the pathogenesis of IDH mutant tumors, as high *CYTOR* expression is associated with poor overall survival. Given our small sample size, we were unable to determine whether chromatin accessibility of CYTOR varied by grade in IDHmut-codel samples. However, our integrative data indicates that a subset of IDHmut-codel tumors also harbors an open chromatin for CYTOR and having an open promoter might be indicative of a malignant subpopulation associated with progression within Grade II IDHmut-codel gliomas. Therefore, it may be possible to target such existing programs at an early stage of tumor development, that could offer a therapeutic benefit for patients. However, future studies are needed to elucidate such interactions during malignant progression.

A limitation of scATAC-seq studies is due to sparsity of data, it is challenging to determine cell identity. Given that IDHmut-codel samples harbor chromosome arm deletions, we were able to infer the presence of 1p/19q deletions in a large majority of our snATAC-seq data. In addition, tumor-associated microglia and macrophages (TAMs) are the most abundant cell types that infiltrate gliomas [9, 43]. However, we did not observe peaks within the promoter for several well-described TAM markers, such as CD11b and CX3CR1, IBA1 or CD45 (data not shown) [1]. Taken together, these results suggest that a large proportion of the signal is obtained from malignant tumor cells.

## Materials and methods

### Patients and tumors

All tumors were obtained from patients following surgical resection at the Department of Neurosurgery at the University Hospital Heidelberg, Germany. Use of patient material was approved by the Institutional Review Board at the Medical Faculty of Heidelberg. Informed consent was obtained from all patients included in the study. Each sample was examined histologically for sufficient tumor cell content of at least 60% and diagnosed by a neuropathologist. The pathologic characteristics of the tumor samples are summarized in Additional file 1: Table S1. RNA-seq data obtained from 693/325 Grade II-IV and 514 Grade II-III glioma samples were downloaded from the Chinese Glioma Genome Atlas (CGGA) and the Cancer Genome Atlas (TCGA), respectively, along with their corresponding clinical information.

### Sample preparation

Following surgical resection, fresh tumor samples were separated into single cell suspensions via mechanical and collagenase enzymatic dissociation (StemCell Technologies- Canada). Debris was removed using tubes with strainer cap (Corning, USA-NY). The cells were snap frozen in a stem cell freezing media (CTS Synth-a-Freeze Medium, Life Technologies) and stored in liquid nitrogen until diagnosis was confirmed.

### Sequencing and quality control

Nuclei were obtained using Nuclei EZ Prep (Sigma-Aldrich, USA-MO). The single nucleus ATAC-Seq protocol performed on HT IFC microfluidics system (Fluidigm, USA-CA) and HiSeq 2000 sequencing technologies (Illumina, USA-CA) with an adapted protocol (Additional file 2: Figure S5). Nuclei were incubated with Tn5 off-chip. The reaction was stopped with EDTA and the nuclei are loaded on the C1 HT IFC. During the amplification in the IFC the first barcode was added to the library which allows pooling per column. After harvesting the PCR products were purified with Ampure beads at a 1.4x ratio. The second barcode was then introduced by a second PCR off-chip and the libraries were size selected with AMPure beads (Beckman Coulter, Brea, CA). Each pool was subjected to quality control using Qubit (ThermoFischer, USA-MA) and TapeStation D1000 (Agilent, USA-CA).

Overall, we tested samples from 7 patient samples, however only 5 samples provided material with sufficient quality for sequencing. One oligodendroglioma sample (NCH5540) was sequenced with 75 bp single reads, while the rest of the samples were sequenced with 50 bp single reads. Duplicate nuclei in C1–800 chip were estimated with visual microscopic analysis, and quality control (QC) was checked at each step. We tested 3 IDHmut-noncodel and 2 IDHmut-codel patient samples. After data processing, 145 cells from IDHmut-noncodel, and 191 cells from IDHmut-codel samples passed our cutoffs with selective peak-calling.

### Computational analysis of snATAC-seq data

#### Data clean-up and alignment

Our initial analysis revealed that some cells have exactly the same read sequences; a phenomenon that cannot be explained by Tn5 enzyme-selectivity alone. The topological spread of the clustered cells revealed that they are mostly located along the same row on the chip, and to a lesser extent along the same columns (Additional file 2: Figure S6-d). These distributed reads are unlikely to originate from individual cells, and potentially indicate to an imperfection within the microfluidics platform. We hypothesize that some wells with a high number of reads spill over to neighboring wells along the microfluidic platform. Target wells are probably empty as they have low number of unique reads with less variability. One explanation for the event might be that wells with duplicated nuclei generate irregular fluid movement that causes asynchronized nuclei lysis or major concentration gradient and differences in liquid densities. This is supported by the fact that the empty chambers are the main target to be filled with the leaking reads. A large part of our analysis focused on cleaning the data and performing quality controls. To handle the well-to-well leakage, we applied the standard de-duplication with Picard’s MarkDuplicate (http://broadinstitute.github.io/picard) but failed to reduce biases due to leakages. We have chosen to remove all reads that exist in more than one well before alignment using custom python3.7 script (described later). Noticeably, fastq files were inflated with PCR amplicons and some read sequences sustained mutations after PCR amplification and sequencing. A small percentage in total, this kept introducing bias to the final result. Ultimately, we deduplicated fastq files using clumpify (sourceforge.net/projects/bbmap/) with default dedupe parameters which removed most of the duplicates and the mutant amplicons. Then, we applied the custom python3.7 script to remove the leaky reads among the cells. The script reads each individual fastq file, iterates through each of the other files in the sample/HT-IFC chip, then removes any duplicated read. The script removed the exact sequence of DNA that exist in two or more wells. Remaining mutant sequences were not addressed due to unreasonable computational costs. The amount of leakages is reduced by 98% (Additional file 2: Figure S6). As expected, reads tend to gather around transcription start site (TSS) (Additional file 2: Figure S1-b). Every analysis that was performed later is done on these cleaned data. De-duplicated files were aligned with bowtie2 [20] with default parameters against GRCh37.p13 (GenCode), sorted, non-standard chromosomes were removed and bam files were indexed using Samtools 1.9 [23]. Finally, the wells were checked for leaky reads in bed files: percentage of reads with the same coordinates were measured with and without the python script (Additional file 2: Figure S5-b).

#### Bioinformatic analysis

To reduce bias, we called peaks in single cells and used published bulk ATAC-Seq for LGG from TCGA as reference [6]. We called peaks with HOMER v4.10 [13], applying makeTagDirectory with keepAll option. Then we used findPeaks with style histone, minDist 10,000 and size 10,000. The peaks were converted to bed format using pos2bed.pl (HOMER) and annotated with annotatePeaks.pl (HOMER) for hg19. We overlapped our snATAC-Seq peaks with the reference peaks. We defined a peak to have was considered when the overlap is at least 50 bp. Peaks with less than 5 cells were excluded, then cells with less than 50 positive peaks. To remove duplicated nuclei, we removed top 5% of the cells by selecting off the cells with more than 3000 peaks. After applying those parameters, we obtained 336 cells and peaks for 4609 genes. Cells were clustered with t-SNE test for 2 dimensions with perplexity = 30 using Rtsne v0.15 [42] with R v3.6.1. We analyzed read distance from Transcription Starting Sites (TSS). The distance forms a peak of high density in all samples, and some minor peaks further (Fig. 1b). We used TSS table based on atacR v0.4.14 [36], and compared the reads from all wells of a psuedo-bulk file using Samtools 1.9, Rsamtools [30] and GenomicRanges 1.36.0 [21]. Reads around TSS in a range of 2 kb (− 2000 to + 2000), are 11.4, 8.8, 5.8, 17.2 and 6.6% for samples astro1, astro2, astro3, oligo1 and oligo2 respectively.

#### Enrichment

Most of the peaks fall into intronic and intergenic annotation. We overlapped the intergenic peaks with miRNA data from USCS and with enhancers from HACER db [44]. The regions from enhancers were reduced using GenomicRanges and overlapped with Homer peaks from each cell. The intergenic peaks that were not classified as miRNA or enhancers were called “others” (Fig. 1d). We evaluated functional cis-regulatory regions for enhancers using Genomic Regions Enrichment of Annotation Tools (GREAT) [29]. We divided the cells into Oligodendrogliomas and Astrocytomas, and we obtained the enhancers that overlapped more than 10 cells. We generated a BED file from the peaks of each group, and we uploaded the file to GREAT website. We used gene ontology for Biological processes with over 4 Binomial Fold Enrichment.

#### Clustering

To identify clusters in our data and to find top expressed genes, we used k-means algorithm (*k* = 5) in R v3.61 base. The clusters were not perfect, so we improved manually the borders of the clusters (Additional file 2: Figure S3). The two branches consist of two clusters each, and the undefined nuclei cluster in the fifth.

Based on the manual clustering, we looked for top expressed genes and top specific genes for each group and each cluster. The equations for those two markers as follows:

$$ TopGenes=\mathrm{sort}\left(\ {\mathrm{x}}^{+}/{\mathrm{Y}}^{+}-\left(\mathrm{x}-+1\right)/{\mathrm{Y}}^{-}\ \right),\mathrm{where}\ {\mathrm{x}}^{+}>10 $$

$$ SpecificGenes=\mathrm{sort}\left(\ {\mathrm{x}}^{+}/{\mathrm{Y}}^{+}/\left(\mathrm{x}-+1\right)/{\mathrm{Y}}^{-}\ \right),\mathrm{where}\ {\mathrm{x}}^{+}>10 $$

x^+^: Number of nuclei with peak(s) for the gene in the specific group/cluster

Y^+^: Total number of nuclei with peak(s) for the gene

x^-^: Number of nuclei with no peaks for the gene in the specific group/cluster

Y^-^: Total number of nuclei with no peaks for the gene

*ATAC-seq coverage and CNV.* We used a method similar to Satpathy et al. [32]. Chromosomes were tiled into 10 Mb regions with 2 Mb overlap (using the bedtools makewindows function), and the average ATAC-seq coverage for each cell was computed inside this window using the bigWigAverageOverBed function. Each window was normalized using 100 windows with matching GC content (excluding windows from the 1p and 19q arm). The normalized coverage of a window *w*_*0*_ was computed as

$$ \mathrm{CovNorm}\left({w}_0\right)=\underset{i=\mathrm{1..100}}{\mathrm{mean}}\log \left(\frac{\operatorname{cov}\ \left({w}_0\right)+1}{\operatorname{cov}\left({w}_i\right)+1}\right) $$

were *w*_*i*_ represent the GC-content matching windows. Telomeric and centromeric regions with low mapping percentage were manually excluded from the plots based on the mapping profiles over the single-cells.

#### Transcription factor activity

We used chromVAR v1.6.0 to infer transcription factor accessibility in our snATAC-seq. Counts were obtained with getCounts function using peaks from bulk ATAC-seq TCGA for LGG (peak-names start with LGG), a CG bias was corrected, and peaks filtered with min_depth = 500 and min_in_peaks = 0.013. A total 280 cells passed the filtering process, which were matched to JasperMotifs [18] and genome from BSgenome.Hsapiens.UCSC.hg19 v1.4. Background peaks were prepared, and deviation was measured via computeDeviations function. Variability was calculated and plotted. deviationsTsne and plotDeviationsTsne were applied for the top ten genes. A heatmap for sample correlation was produced by the same tool with threshold of 1 (Additional file 2: Figure S4). The difference between number of cells that passed the chromVAR criteria and those that passed our method can be attributed to the differences in the cutoff set by the method.

#### Web-based explorer for data visualization

We used the peaks we called and selected against bulk TCGA ATAC-Seq peaks as starting point to produce a web-based explorer of the data. Gene annotation were used from TCGA publication and all peaks related to one gene were collapsed after t-SNE clustering. A SHINY-R3.6 app (cran.r-project.org) was developed with ggplot2 library (H. Wickham. ggplot2: Elegant Graphics for Data Analysis. Springer-Verlag New York, 2016.) and tested on amazon cloud service (*AWS-EC2*) with 1GB of RAM and 1 core on Ubuntu 18 server instance, and a duplicate copy was tested on a similar server from *hostinger.com* to check platform compatibility. To visualize the data from selective peak-calling, we produced an explorer based on R/ shiny package 1.3.2. The clustered data, the code and a manual are submitted at github.com/RuslanAlali/SHINY_scATAC-Seq. Data can be visualized at shiny.turcanlab.org.

### Array-based methylation analysis

Methylation analysis was performed using the Illumina HumanMethylationEPIC bead array at Heidelberg Neuropathology. R statistical software (v3.6.0) was used for data analysis. The minfi methylation pipeline was used to extract and analyze data from idat files, and normalization was carried out using functional normalization method [11]. Differentially methylated positions between IDHmut-codel and IDHmut-noncodel gliomas were detected using dmpFinder in minfi package. Loci with q-value < 0.001 and absolute β-value > 0.1 were considered to be differentially methylated.

### CGGA RNA-seq data analysis

CGGA RNA-seq datasets were used to determine the Pearson’s correlation coefficient for *CYTOR* versus all other genes. Genes with correlation coefficients > 0.5 were considered to be positively correlated with *CYTOR.* To determine the functional enrichment of genes correlating with *CYTOR*, WebGestalt was used to perform over-representation using the following functional databases: Gene Ontology databases (molecular function, biological process), and Pathway (KEGG, and REACTOME) [24].

### Statistical analysis

All statistical analyses were performed using the R software (v3.6.0). RNA-seq data from the CGGA cohort was downloaded, and processed in R. The maxstat R package was used to determine the optimal cut-off points for *CYTOR* expression to dichotomize patients into low and high expression groups [15]. The survival and survminer R packages were used for Kaplan-Meier analysis to estimate the survival curves of IDHmut-codel and IDHmut-noncodel subgroups [17, 38]. Statistical comparison of groups was calculated using the log rank test. *P*-value less than 0.05 was considered statistically significant.

### Data availability

Called peaks from Homer pipeline have been deposited in the Gene Expression Omnibus under accession number GSE137266.

## Conclusion

In conclusion, although we obtained a limited number of nuclei from five samples, scATAC-seq is shown to be scalable to scRNA-seq level with thousands of cells [3]. By integrating the snATAC-seq data with unbiased data analysis from bulk methylation, RNA-seq and ATAC-seq datasets, we were able to provide initial insights into glioma epigenetics at the level of individual nuclei. As new high-throughput single cell ATAC-seq technologies have become available, we envision that future studies will further expand our understanding of the epigenetic order in gliomas.

## Supplementary information

**Additional file 1: Table** **S1.** Statistics on snATAC-seq data.

**Additional file 2: Figure S1.** The effect of removal of leaky reads from Oligo1. **Figure S2.** Different clustering approaches on snATAC-seq data. **Figure S3.** Cluster identification. **Figure S4.** chromVAR results on all 5 samples. **Figure S5.** Overview of snATAC workflow. **Figure S6.** Prognostic significance of CYTOR in IDH mutant gliomas.

**Additional file 3: Table S2.** chromVAR analysis of snATAC-seq data indicates variability scores for 386 transcription factor motifs.

**Additional file 4: Table S3.** Genes that correlate with specific open chromatin in IDH mutant LGGs.

**Additional file 5: Table S4.** Regions correlating with CYTOR across all samples.

**Additional file 6: Table S5.** Differentially methylated probes between IDH-codel and IDH-intact samples (q-value < 0.001 and absolute delta ß-value > 0.1).

**Additional file 7: Table S6.** Genes correlating with CYTOR in the CGGA cohorts.

**Additional file 8: Table S7.** Overrepresented gene sets in the 101 genes correlating with CYTOR.
