# Supplementary material for: Single-nucleus chromatin accessibility reveals intratumoral epigenetic heterogeneity in IDH1 mutant gliomas
Source: Acta Neuropathol Commun. 2019 Dec 5;7:201. doi: 10.1186/s40478-019-0851-y (PMC6896263; doi:10.1186/s40478-019-0851-y)
Supplement: Supplementary file 2 — Additional file 2: Figure S1. The effect of removal of leaky reads from Oligo1. Figure S2. Different clustering approaches on snATAC-seq data. Figure S3. Cluster identification. Figure S4. chromVAR results on all 5 samples. Figure S5. Overview of snATAC workflow. Figure S6. Prognostic significance of CYTOR in IDH mutant gliomas. [file 40478_2019_851_MOESM2_ESM.pptx]

## Slide 1
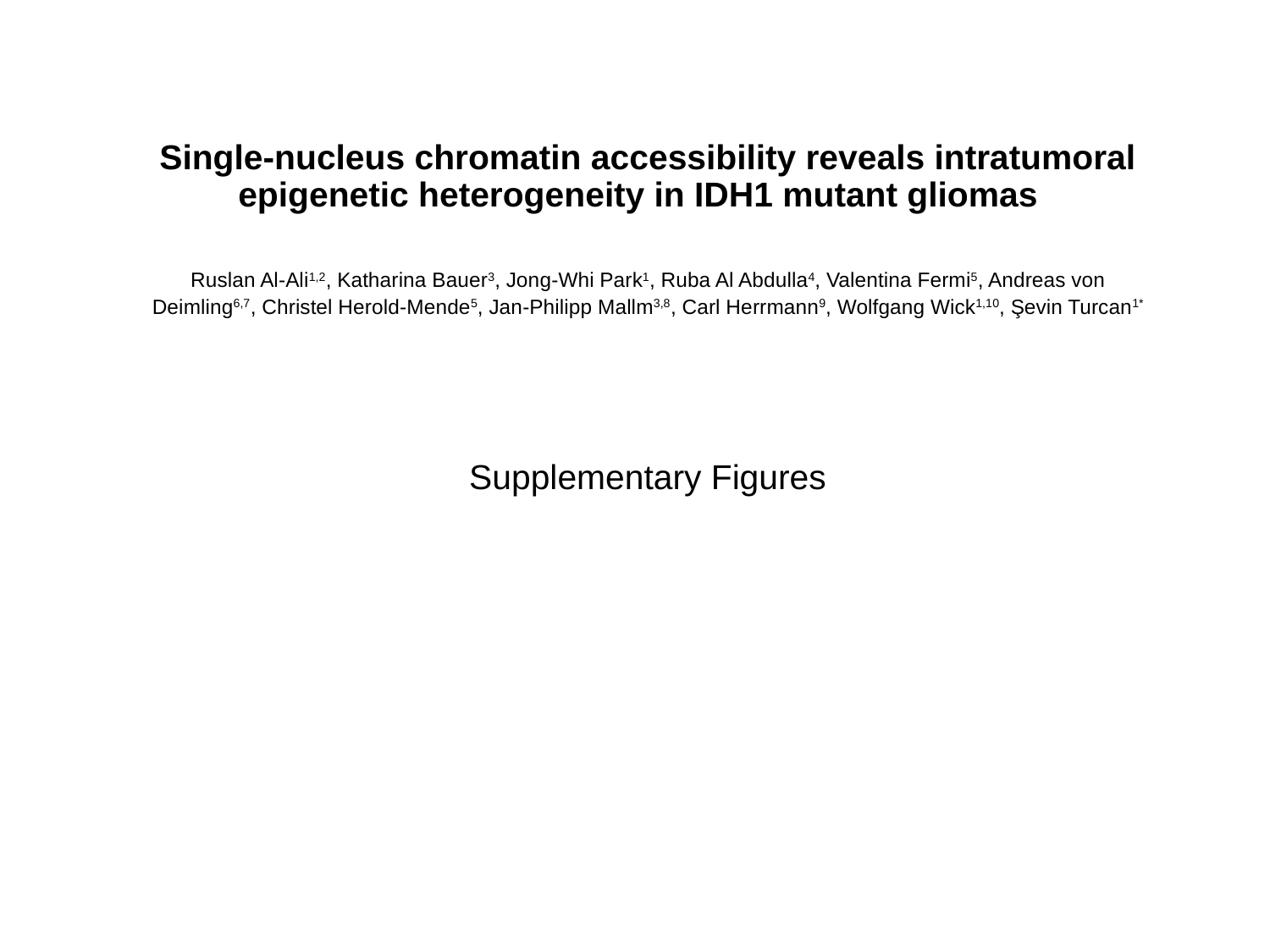

Single-nucleus chromatin accessibility reveals intratumoral epigenetic heterogeneity in IDH1 mutant gliomas
Ruslan Al-Ali1,2, Katharina Bauer3, Jong-Whi Park1, Ruba Al Abdulla4, Valentina Fermi5, Andreas von Deimling6,7, Christel Herold-Mende5, Jan-Philipp Mallm3,8, Carl Herrmann9, Wolfgang Wick1,10, Şevin Turcan1*
Supplementary Figures

## Slide 2
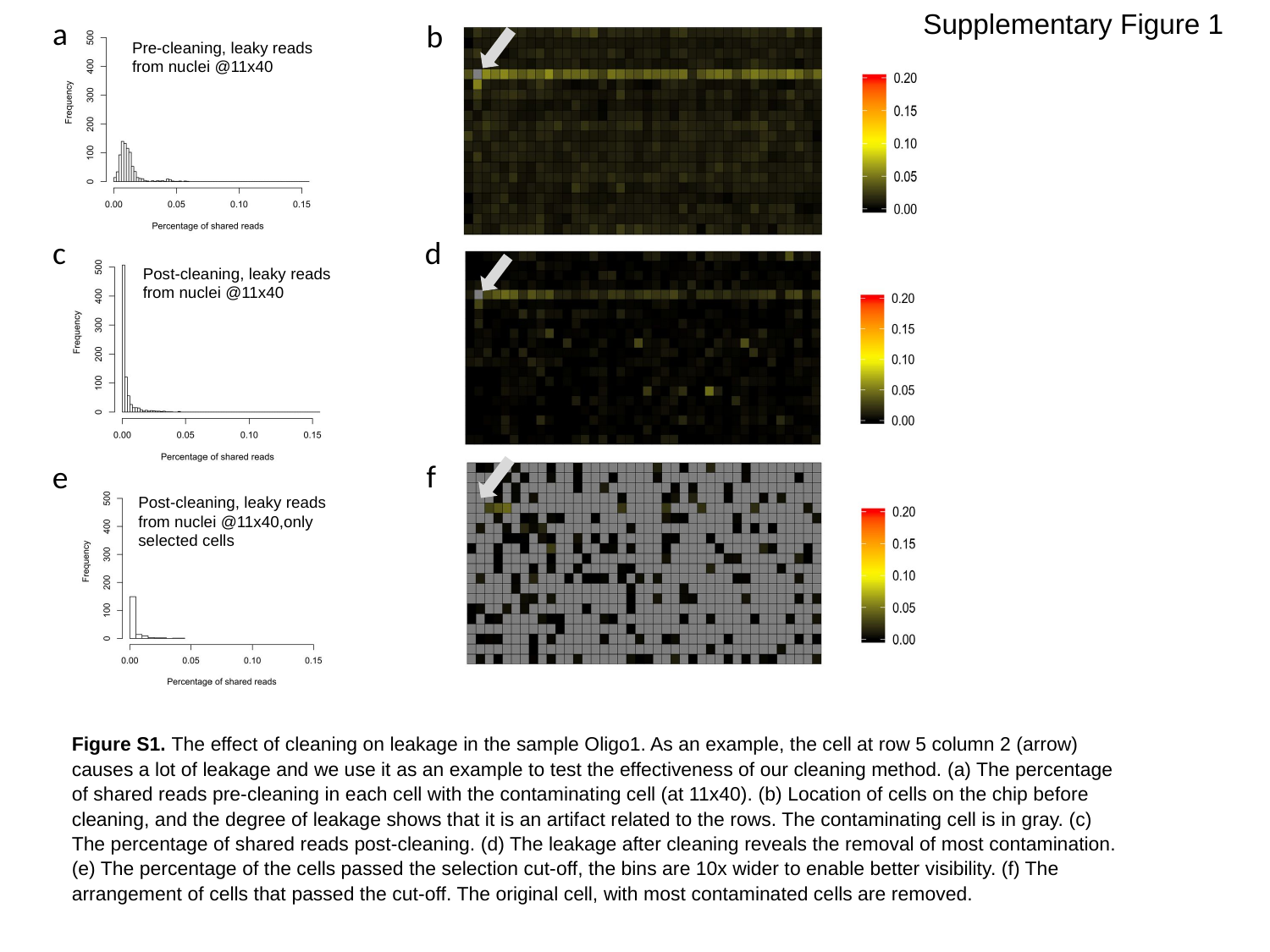

Supplementary Figure 1
a
b
Pre-cleaning, leaky reads from nuclei @11x40
c
d
Post-cleaning, leaky reads from nuclei @11x40
f
e
Post-cleaning, leaky reads from nuclei @11x40,only selected cells
Figure S1. The effect of cleaning on leakage in the sample Oligo1. As an example, the cell at row 5 column 2 (arrow) causes a lot of leakage and we use it as an example to test the effectiveness of our cleaning method. (a) The percentage of shared reads pre-cleaning in each cell with the contaminating cell (at 11x40). (b) Location of cells on the chip before cleaning, and the degree of leakage shows that it is an artifact related to the rows. The contaminating cell is in gray. (c) The percentage of shared reads post-cleaning. (d) The leakage after cleaning reveals the removal of most contamination. (e) The percentage of the cells passed the selection cut-off, the bins are 10x wider to enable better visibility. (f) The arrangement of cells that passed the cut-off. The original cell, with most contaminated cells are removed.

## Slide 3
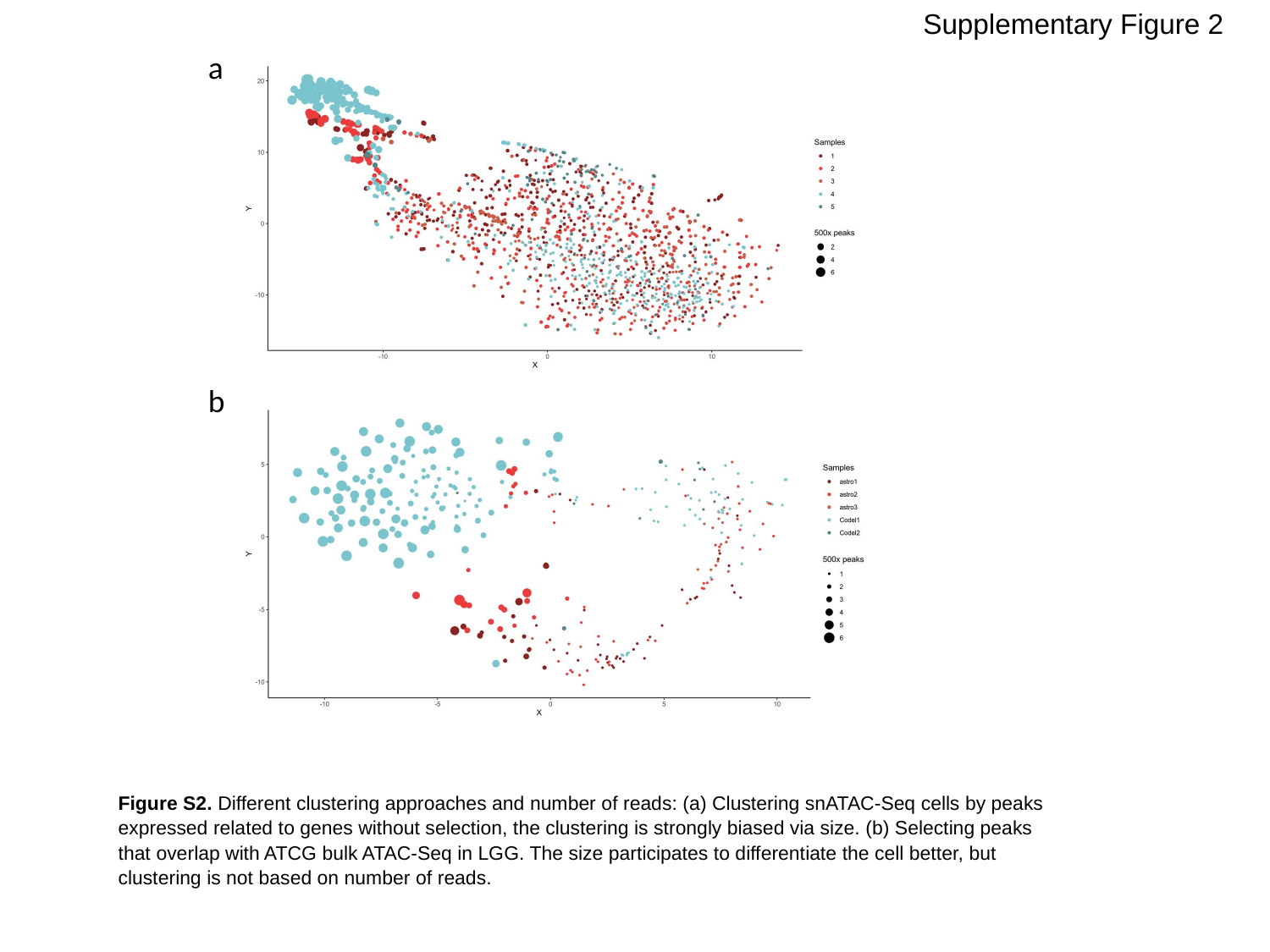

Supplementary Figure 2
a
b
Figure S2. Different clustering approaches and number of reads: (a) Clustering snATAC-Seq cells by peaks expressed related to genes without selection, the clustering is strongly biased via size. (b) Selecting peaks that overlap with ATCG bulk ATAC-Seq in LGG. The size participates to differentiate the cell better, but clustering is not based on number of reads.

## Slide 4
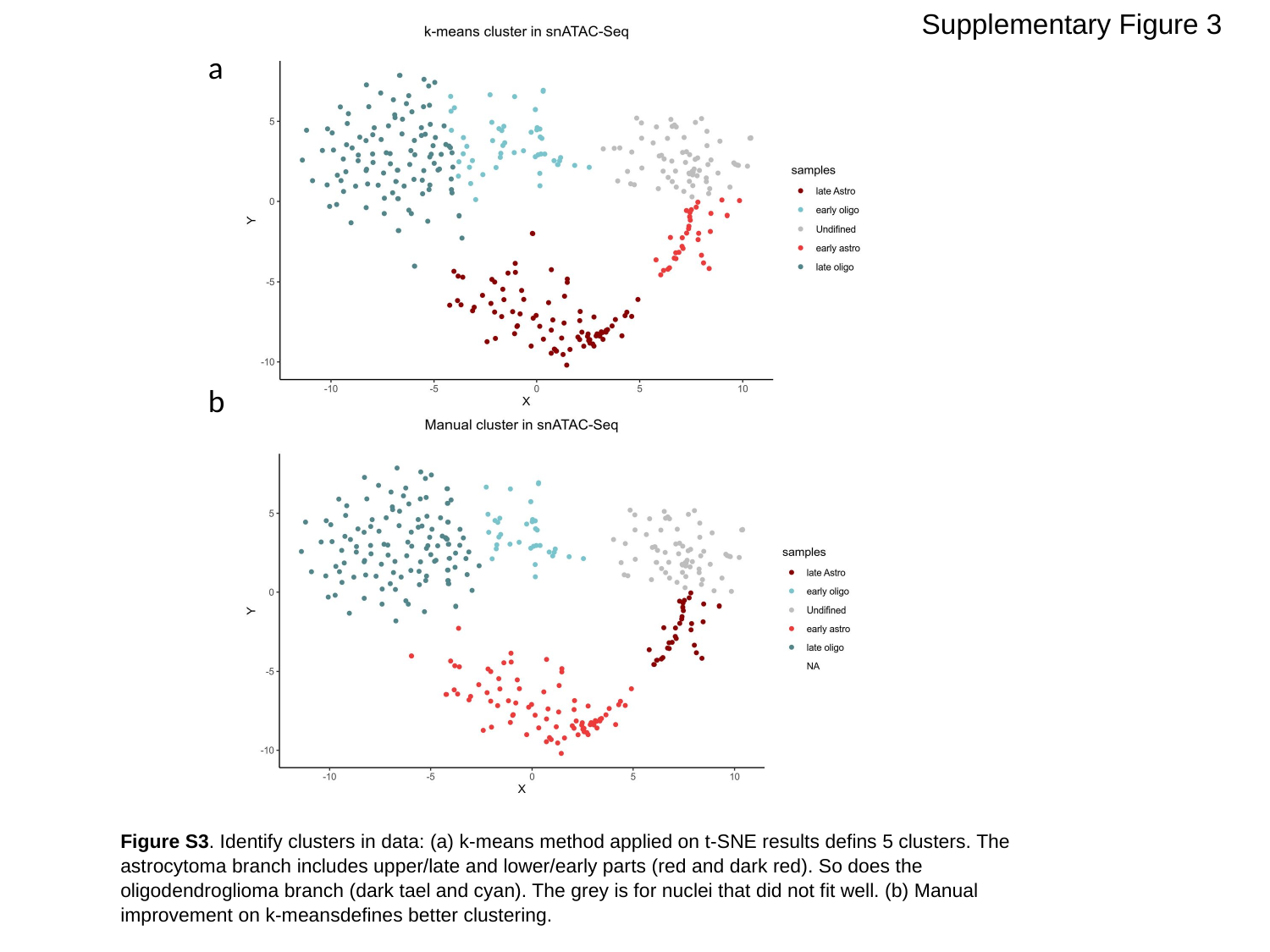

Supplementary Figure 3
a
b
Figure S3. Identify clusters in data: (a) k-means method applied on t-SNE results defins 5 clusters. The astrocytoma branch includes upper/late and lower/early parts (red and dark red). So does the oligodendroglioma branch (dark tael and cyan). The grey is for nuclei that did not fit well. (b) Manual improvement on k-meansdefines better clustering.

## Slide 5
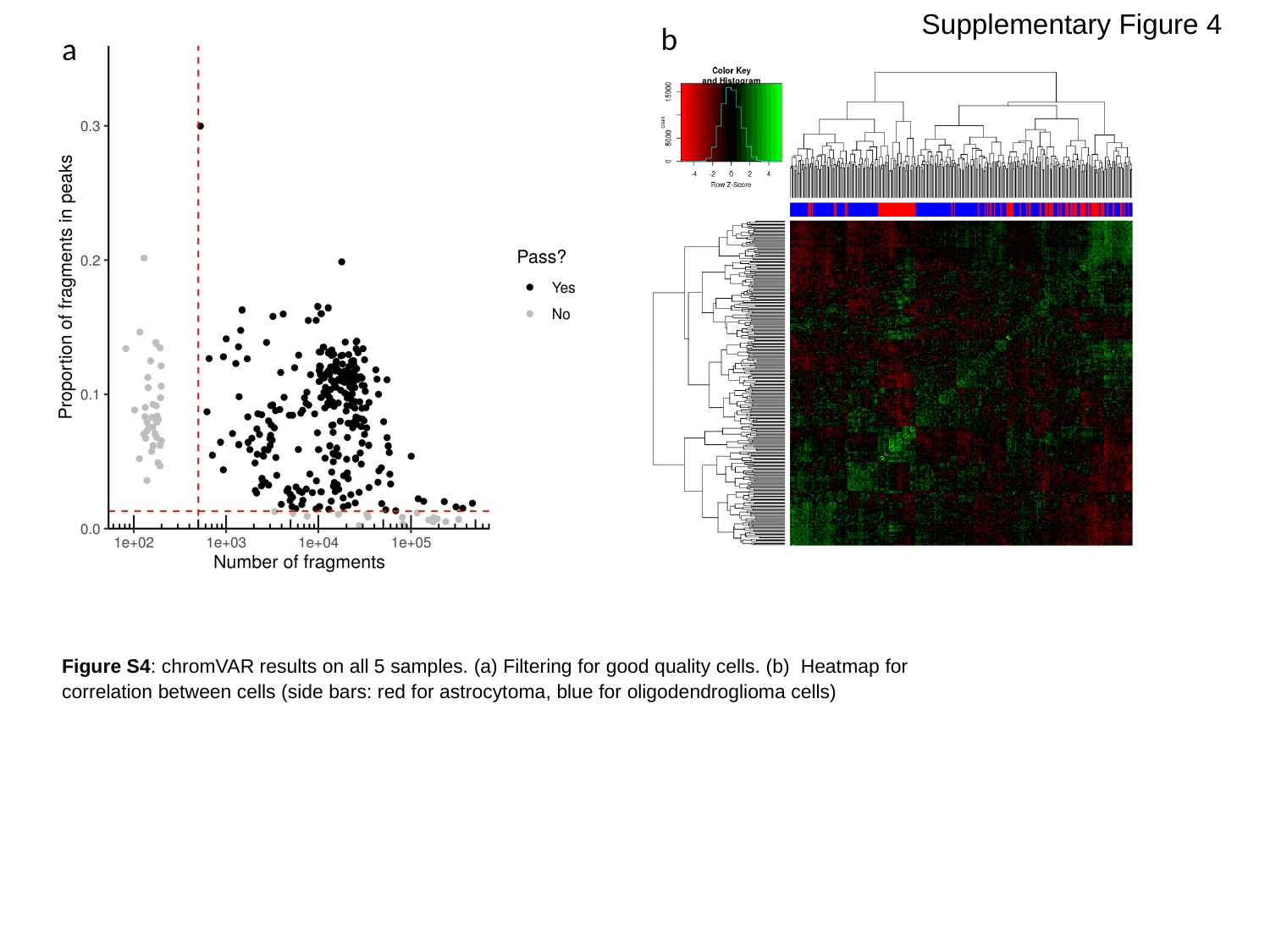

Supplementary Figure 4
b
a
Figure S4: chromVAR results on all 5 samples. (a) Filtering for good quality cells. (b) Heatmap for correlation between cells (side bars: red for astrocytoma, blue for oligodendroglioma cells)

## Slide 6
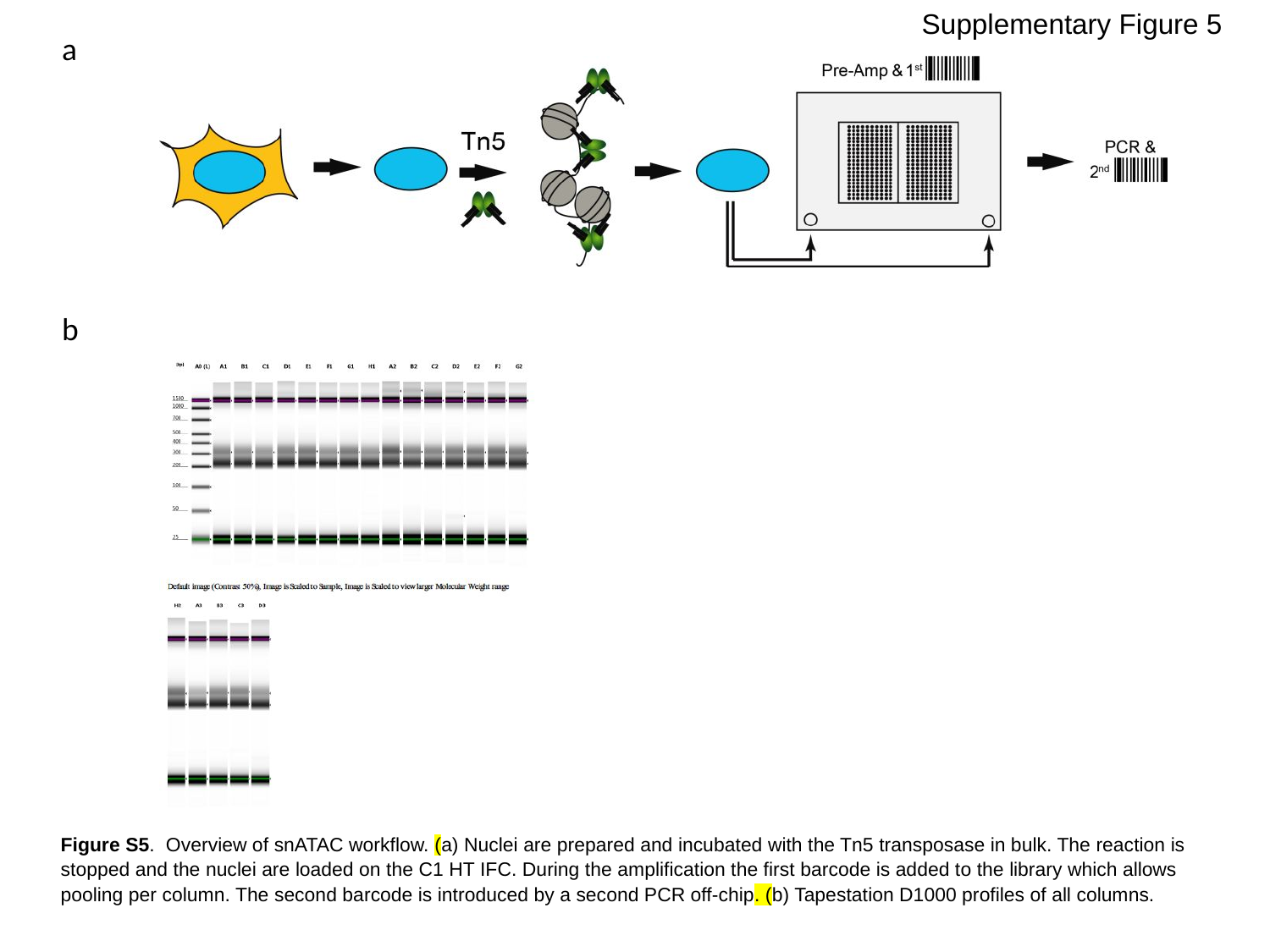

Supplementary Figure 5
a
b
Figure S5. Overview of snATAC workflow. (a) Nuclei are prepared and incubated with the Tn5 transposase in bulk. The reaction is stopped and the nuclei are loaded on the C1 HT IFC. During the amplification the first barcode is added to the library which allows pooling per column. The second barcode is introduced by a second PCR off-chip. (b) Tapestation D1000 profiles of all columns.

## Slide 7
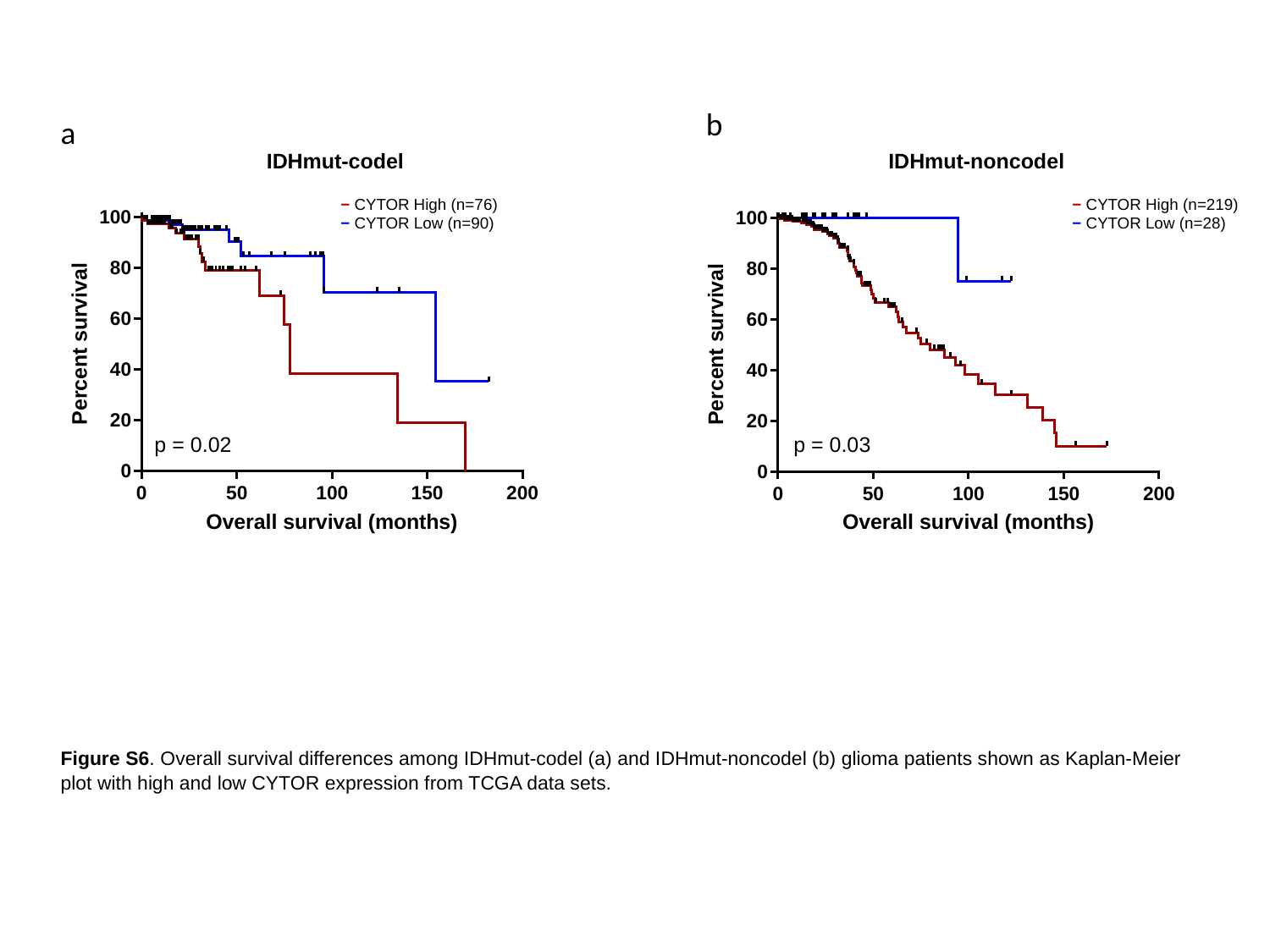

b
a
IDHmut-noncodel
IDHmut-codel
− CYTOR High (n=76)
− CYTOR Low (n=90)
− CYTOR High (n=219)
− CYTOR Low (n=28)
p = 0.02
p = 0.03
Figure S6. Overall survival differences among IDHmut-codel (a) and IDHmut-noncodel (b) glioma patients shown as Kaplan-Meier plot with high and low CYTOR expression from TCGA data sets.
